# Simulation of an Adaptive Fluid-Membrane Piezoelectric Lens

**DOI:** 10.3390/mi10120797

**Published:** 2019-11-20

**Authors:** Hitesh Gowda Bettaswamy Gowda, Ulrike Wallrabe

**Affiliations:** Laboratory for Microactuators, IMTEK - Department of Microsystems Engineering, University of Freiburg, Georges-Koehler-Allee 102, Room 02-081, 79110 Freiburg, Germany; hitesh.gowda@imtek.uni-freiburg.de

**Keywords:** adaptive lens, piezoelectric devices, fluid-structure interaction, moving mesh, thermal expansion, COMSOL

## Abstract

In this paper, we present a finite-element simulation of an adaptive piezoelectric fluid-membrane lens for which we modelled the fluid-structure interaction and resulting membrane deformation in COMSOL Multiphysics^®^. Our model shows the explicit coupling of the piezoelectric physics with the fluid dynamics physics to simulate the interaction between the piezoelectric and the fluid forces that contribute to the deformation of a flexible membrane in the adaptive lens. Furthermore, the simulation model is extended to describe the membrane deformation by additional fluid forces from the fluid thermal expansion. Subsequently, the simulation model is used to study the refractive power of the adaptive lens as a function of internal fluid pressure and analyze the effect of the fluid thermal expansion on the refractive power. Finally, the simulation results of the refractive power are compared to the experimental results at different actuation levels and temperatures validating the coupled COMSOL model very well. This is explicitly proven by explaining an observed positive drift of the refractive power at higher temperatures.

## 1. Introduction

The expression adaptive optics was initially termed for the technology used in telescopes to deform the mirrors for phase correction of the incoming light [1]. Soon, adaptive optics was implemented in microscopes [2], optical communication systems [3], and optical imaging systems [4]. In conventional imaging systems, the lenses are mechanically moved to focus an image, whereas, with the adaptive optics lens, the surface curvature of the lens is changed to focus an image. The tunable focus lens, also known as the adaptive lens, uses different actuation principles to change the curvature of a deformable surface, thereby changing the focus (refractive power) of the lens. One such adaptive lens using a piezoelectric actuation principle to deform a fluid-membrane interface [5] was developed in the Laboratory for Microactuators, IMTEK - Department of Microsystems Engineering, University of Freiburg, Germany.

**Figure 1:**
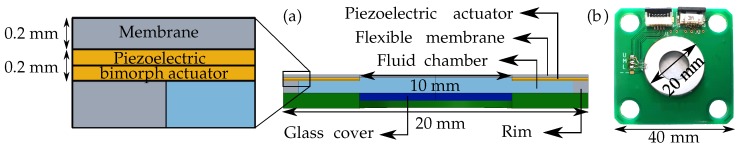
(**a**) 2D cut section of the adaptive lens to show the fluid chamber, flexible membrane, and the integrated actuator. (**b**) The adaptive fluid-membrane piezoelectric lens.

The developed adaptive lens consists of a piezoelectric actuator, a fluid chamber, and a transparent flexible membrane, as shown in Figure 1a. The flexible membrane bounds the fluid chamber on one side, and hence any change in the fluid chamber pressure will deform the membrane. An electric field applied on the piezoelectric actuator will deform the fluid chamber and change the fluid chamber pressure. By changing the electric field direction and magnitude, the fluid pressure can be varied to positive or negative pressures resulting in a varied refractive power. The refractive power defined as a function of the applied electric field exhibits piezoelectric hysteresis [6]. At higher temperatures, the fluid expansion will also contribute to the membrane deformation [7]. The piezoelectric hysteresis and thermal expansion contribute to a non-linear response of the adaptive lens. As the membrane deformation is a direct result of the fluid pressure change, the non-linear effects can be addressed by defining the refractive power as a function of the fluid pressure.

To address the non-linear response and hence to compensate for the hysteresis and the temperature effect on the refractive power, it is essential to determine the combined influence of piezoelectric actuation and temperature on the membrane deformation. Hence in this paper, we present a finite-element simulation of the adaptive lens modelled in COMSOL Multiphysics^®^ (5.3a, COMSOL Inc, Burlington, MA, USA) to define the refractive power linearly as a function of both the fluid pressure and temperature.

COMSOL Multiphysics^®^ is based on the finite-element method (FEM), which solves engineering problems such as structural mechanics, fluid dynamics, heat transfer by a numerical approach. In FEM, the complex geometry is divided into simpler domains. These domains are defined with the elementary partial differential equations based on the physics. Then the elementary equations are combined to form a system of global equations, which represent the complex geometry [8]. The system of global equations can be solved using FEM-based simulation software such as ANSYS, ABAQUS, ATILA, and COMSOL [9]. To simulate complex geometry with multiple physics domains, COMSOL Multiphysics^®^ offers a methodological environment to access elementary equations and then couple them with a wide range of available physics modules [10]. Using COMSOL^®^, authors in [11,12,13] simulated adaptive lenses using only the piezoelectric physics module, authors in [14,15] simulated micro-pumps using the fluid-structure interaction physics module and authors in [16,17] simulated thermal actuators using the heat transfer physics module. However, the articles [11,12,13,14,15,16,17] did not simulate any kind of solid deformation produced by the fluid forces from both the piezoelectric actuation and the thermal expansion. Furthermore, COMSOL^®^ does not provide a direct feature to couple the piezoelectric with the fluid-structure and heat transfer physics modules. Hence, in this paper, we present the explicit coupling of multiple physics modules to simulate the membrane deformation due to the fluid forces from both the piezoelectric actuation and the thermal expansion.

We describe the physical design and working principle of the adaptive lens in Section 2. In Section 3, we describe the simulation model of the adaptive lens and describe the explicit coupling of multiple physics modules using a moving mesh physics module. In Section 4, we present the simulation results of the adaptive lens and compare the simulation results with the experimental results in Section 5. We conclude our paper with results in Section 6.

## 2. The Fluid-Membrane Piezoelectric Lens

The piezoelectric bi-morph actuator has a circular ring-shaped design with the two piezoelectric ceramic layers glued together in an anti-parallel polarization configuration. The fluid chamber and the flexible membrane are integrated with the actuator using micro-molding techniques to form an active lens chamber [5]. The active lens chamber is glued onto a PCB-based substrate [7] and primed with an optical oil [7] (Figure 1a).

**Figure 2:**
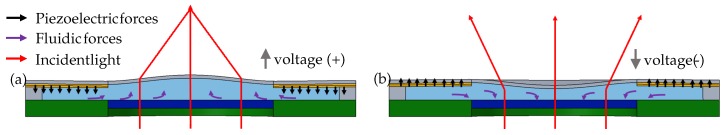
The 2D cross-section of the adaptive lens showing the piezoelectric forces and fluidic forces, which form either (**a**) plano-convex lens or (**b**) plano-concave lens depending on the applied voltage direction.

The manufactured adaptive lens with the actuator diameter of 20 mm with an aperture of 10 mm is shown in Figure 1b. The adaptive lens has an overall thickness of around 2.2 mm, with substrate thickness of 1 mm, rim thickness of 0.8 mm, membrane thickness of 0.2 mm and bi-morph actuator thickness of 0.2 mm. Depending on the applied electric field/voltage direction, the actuator deforms the fluid chamber to produce positive or negative fluid chamber pressure. The positive pressure leads to a plano-convex lens (Figure 2a), and the negative pressure leads to a plano-concave lens (Figure 2b).

## 3. Multiphysics Simulation

**Figure 3:**
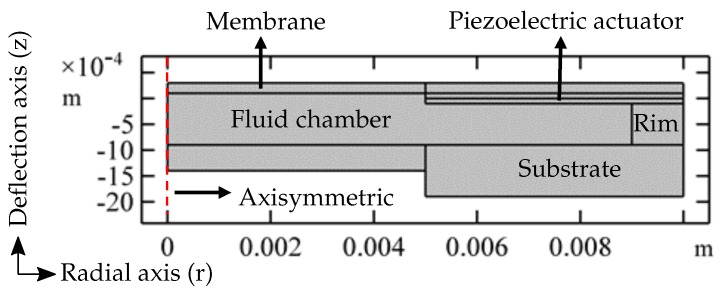
The 2D axisymmetric simulation model of the adaptive lens.

To reduce the complexity of a 3D geometry and at the same time to decrease the computation time, a 2D-axisymmetric space dimension is chosen to model the adaptive lens (Figure 3) with the radial axis ‘r’ and the deformation axis ‘z’. The adaptive lens components include a piezoelectric actuator, fluid, membrane, rim, and substrate. The materials for the components are chosen from the COMSOL Multiphysics^®^ inbuilt material library [18]. The material parameters are changed to the equivalent parameters of the materials, which are used in the manufacture of the adaptive lens. The adaptive lens components, along with the modified material parameters used in the simulation, are mentioned in Table 1 and the adaptive lens components thicknesses are mentioned in Table 2. The following section describes the physics modules used in the simulation.

### 3.1. Piezoelectric Devices

The adaptive lens uses the inverse piezoelectric property of the actuator to vary the refractive power. To model the inverse piezoelectric effect, the piezoelectric devices module is used. The module couples the solid mechanics Equation (1) and the electrostatics Equation (2) physics to combine the electrical behavior and the mechanical behavior of the piezoelectric ceramics.

(1) $$\rho \frac{\partial^{2}x}{\partial^{2}t}=▿\;\cdot \;s+F_{v}$$

where ρ is the density, *x* is the displacement, *s* is the stress, and $F_{v}$ is the volume force.

(2) E=−▿·V

where *E* is the electric field, and *V* is the electric potential.

The combined behavior is modelled through the coupled Equations (3) and (4) in strain-charge form.

(3) $$S=S_{E}T+d^{T}E$$

(4) $$D=dT+\epsilon_{o}\epsilon_{rT}E$$

where the solid mechanics parameters are strain *S* and stress *T*, the electrostatic parameters are electric field *E* and electric displacement field *D*, and the piezoelectric material parameters are compliance coefficient $S_{E}$, piezoelectric coefficient $d^{T}$, and permittivity ϵ [23]. In the simulation model, the piezoelectric coefficients and compliance coefficients are obtained from the piezo PZT-5H inbuilt material library [18].

### 3.2. Fluid-Structure Interaction

The piezoelectric actuator in the adaptive lens deforms the fluid chamber and varies the internal fluid pressure. The varied internal fluid pressure results in fluid forces, which act on the flexible membrane. The fluid forces contribute to the deformation of the flexible membrane to an aspherical surface. The fluid-structure interaction (FSI) physics module models the fluid forces acting on the membrane by coupling the solid mechanics Equation (1) and the laminar flow Equation (5) physics modules.

(5) $$\rho \frac{\partial u}{\partial t}+\rho \left(u\;\cdot \;▿\right)u=\mu {▿}^{2}u+F+\rho g$$

The Navier–Stokes Equation (5) models the motion of incompressible fluids, where ρ is the fluid density, *u* is the fluid velocity, *F* is the external force and *g* is the gravity. The FSI Multiphysics module couples the fluid inertial forces in Equation (5) with the external forces in Equation (1) [24].

### 3.3. Heat Transfer in Solids and Fluids

Apart from the piezoelectric actuation that contributes to the deformation of the membrane, the thermal expansion of the fluid at higher temperatures will as well cause the membrane deformation. Hence to model the fluid thermal expansion, heat transfer in solids (Equation (6)) and heat transfer in fluids (Equation (7)) physics modules are used.

(6) $$Q=\alpha T\;\cdot \;\frac{dS}{dt}$$

(7) $$Q=\alpha T(\frac{\partial \rho }{\partial t}+u\;\cdot \;▿p)$$

where *Q* is the heat source, *T* is the temperature, *S* is the solid stress tensor, α is the coefficient of thermal expansion, *p* is the fluid pressure, and *u* is the fluid velocity.

Equations (6) and (7) define the heat source *Q* that contributes to set the complete adaptive lens domain to the required temperature *T*. Equation (7) models the thermal expansion that contributes to the fluid pressure *p*, which acts on the flexible membrane [25].

### 3.4. Moving Mesh

**Figure 4:**
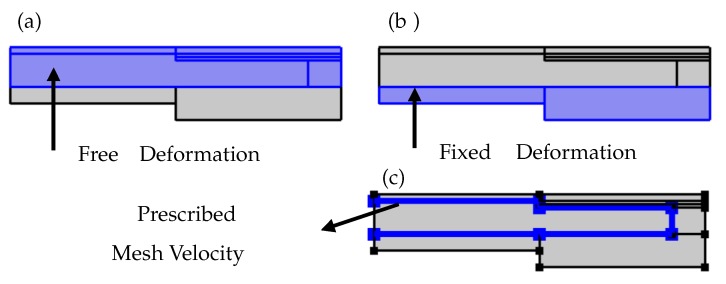
The domains specified in the Moving Mesh module to be (**a**) free mesh and (**b**) fixed mesh. (**c**) The solid domain velocity applied on the walls of the fluid chamber.

The adaptive lens working mechanism relies on the transfer of piezoelectric forces to the flexible membrane through the fluid forces. In COMSOL Multiphysics^®^, the coupling of the piezoelectric effect and the fluid-structure interaction is not possible through a direct Multiphysics feature. Hence, the moving mesh physics module is used to couple the piezoelectric forces with the fluid forces and apply the resultant on the flexible membrane. The explicit coupling of piezoelectric and laminar flow physics is performed in a way such that the solid domain velocities Equations (8) and (9) generated by the deformation of the piezoelectric actuator are applied as the mesh velocities on the walls of the fluid chamber as shown in Figure 4c. The geometric domains with the free deformation mesh and with the fixed mesh are as shown in Figure 4a,b, respectively.

(8) $$V_{r}=solid\;\cdot \;u\_tR$$

(9) $$V_{z}=solid\;\cdot \;u\_tZ$$

Equations (8) and (9) equate the solid velocities u_tR and u_tZ, which are produced by the piezoelectric actuator in radial axis (R) and deformation axis (Z), to the corresponding mesh velocities $V_{r}$ and $V_{z}$. The mesh velocities applied to the fluid chamber wall are implicitly considered to be the external forces in the Navier–Stokes Equation (5) of the fluid-structure interaction physics module Equation (4). In this way, the moving mesh module explicitly couples the piezoelectric actuator force to the fluid force.

### 3.5. Boundary Condition

**Figure 5:**
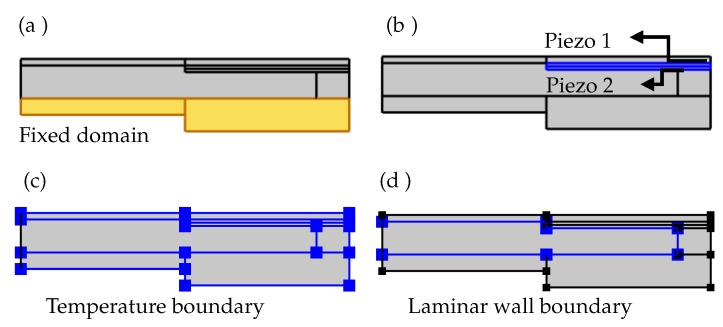
Domains with fixed boundary condition in solid mechanics physics module (**a**), piezo 1 with default base vector system and piezo 2 with modified base vector system (**b**), domains with temperature boundary condition in heat transfer in solids and fluids physics modules (**c**), and domains with wall boundary condition in laminar flow physics module (**d**).

In the solid mechanics interface, the glass substrate is selected in the fixed constraint condition, as shown in Figure 5a. The piezo ceramic layers in the bi-morph actuator have an anti-parallel polarization configuration. To adapt for the polarization direction in the simulation, an additional base vector system is created with the base vector ‘x3’ set to −1 instead of default 1. The default base vector system is selected as the coordinate system for the piezo 1, and the additional base vector system is selected as the coordinate system for the piezo 2 (Figure 5b). In the heat transfer interface, all the boundaries are selected in the temperature constraint, to set the entire domain to the required temperature (Figure 5c). In the laminar flow interface, the walls of the fluid chamber are selected with a no-slip boundary condition (Figure 5d). Since the fluid chamber is a closed domain, there is no inlet, outlet, or open boundary conditions.

### 3.6. Mesh

**Figure 6:**
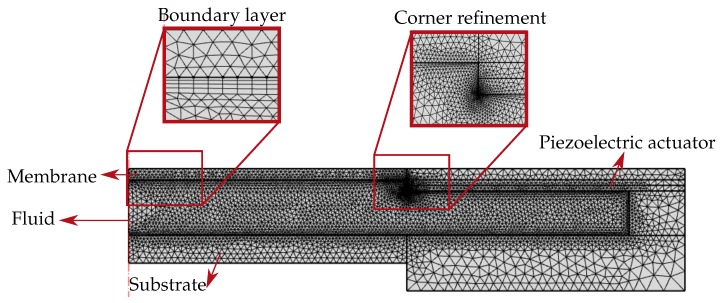
The adaptive lens meshed finely with corner refinement and boundary layer features.

The explicit coupling of the piezoelectric forces with the fluidic forces is achieved through the mesh velocities applied on the walls of the fluid chamber (Figure 4c). Hence the walls of the fluid chamber are finely meshed with the use of corner refinement and boundary layer mesh features (Figure 6). The remaining domains including fluid, piezoelectric actuator, membrane, and the substrate are meshed with the inbuilt physics-controlled mesh.

## 4. Results

**Figure 7:**
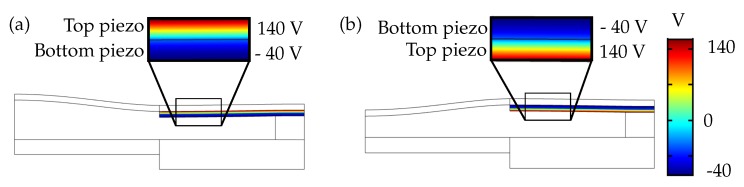
Applied voltages on the piezoelectric actuator with (**a**) convex lens mode and (**b**) concave lens mode.

The time-dependent study was selected in the simulation as the velocities, which are used in the moving mesh module, are time-dependent values, and as the stationary study does not compute these instantaneous velocities. In the study, the time range was selected from 0 s to 1 s with a step of 0.1 s. The applied voltages were limited to 140 V in positive polarization direction, and −40 V in negative polarization direction to avoid piezo saturation and re-polarization [19]. A sinusoidal voltage within the voltage limits was defined using a piecewise function under global definitions. Figure 7 shows the line plot of voltages applied to the piezoelectric actuator.

Figure 7a shows the top piezo set to positive voltage limit of 140 V, and the bottom piezo set to negative voltage limit of −40V, the corresponding voltage combination results in a plano-convex lens. In contrast, the voltage combination is reversed in Figure 7b, i.e., the top piezo is set to −40V and the bottom piezo is set to 140 V, resulting in a plano-concave lens.

**Figure 8:**
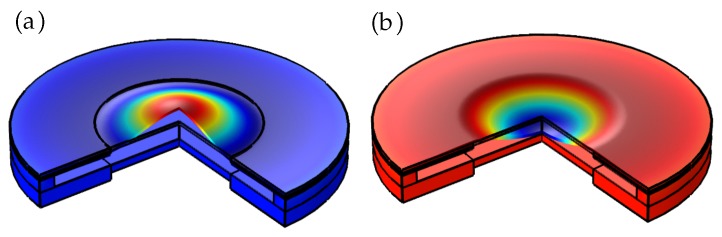
The 2D revolved plots showing (**a**) aspheric convex lens and (**b**) aspheric concave lens mode.

The volume plots in Figure 8a,b generated by the 2D revolution around the symmetric axis show the adaptive lens in plano-convex and plano-concave lens modes. The revolved plot is used to visualize the aspherical deformation of the membrane.

**Figure 9:**
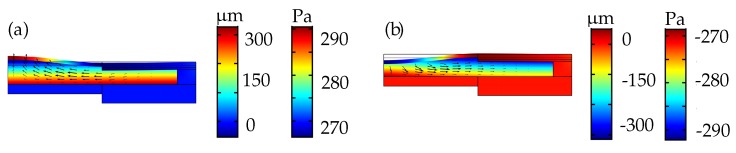
The surface simulation plots of the adaptive lens showing dynamic internal chamber pressure and solid deformation in (**a**) plano-convex and (**b**) plano-concave modes.

The surface plots in Figure 9a,b show the membrane deformation and the corresponding dynamic internal fluid pressure during actuation. The arrows indicate the fluid velocity direction during actuation. For the plano-convex lens in Figure 9a, in which the piezoelectric actuator is set to a maximum voltage combination results in positive internal pressure of around 270 Pa and a peak deflection of around 300 μm at the center of the membrane. For the plano-concave lens in Figure 9b, in which the voltage combination is reversed compared to the former case, the actuator deforms outwards resulting in negative fluid pressure of around −270 Pa and a peak deflection of around −300 μm at the center of the membrane.

**Figure 10:**
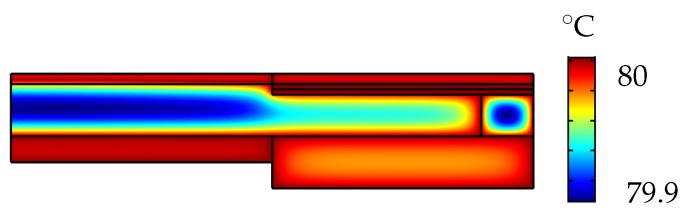
Temperature distribution of the adaptive lens at 80 °C.

The heat transfer interface for solids and fluids was used to set the adaptive lens to a temperature ranging from 20 °C–80 °C. The surface plot in Figure 10 shows the temperature distribution of the adaptive lens set to 80 °C with the actuator voltage set to 0 V. The expansion of the fluid at 80 °C corresponds to an increase in fluid pressure of around 85 Pa and a peak deflection of around 80 μm at the center of the membrane.

**Figure 11:**
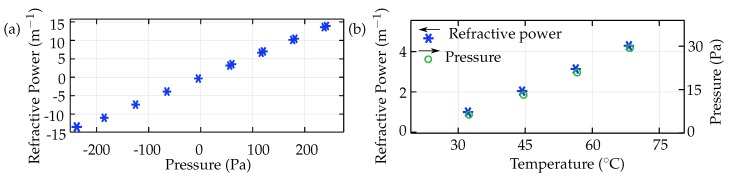
(**a**) The simulated refractive power of the adaptive lens as a function of fluid chamber pressure, and (**b**) the change in refractive power of the adaptive lens due to thermal expansion of the fluid.

The refractive power of the adaptive lens in the simulation (Figure 11) was calculated by double differentiation of the membrane boundary with respect to the deformation component (w) and the radial component (r) Equation (10). Figure 11a shows the refractive power defined as a function of internal fluid pressure. The simulation of the adaptive lens result in a refractive power range of $-16m^{-1}$ to $16m^{-1}$ for an internal fluid pressure range of −270 Pa to +270 Pa.

(10) $$Refractive\;Power=\left(\frac{d^{2}w}{dr^{2}}\right)\;\cdot \;\left(n-1\right)$$

where ‘w’ is the deflection component, ‘r’ is the radial component and ‘n’ is the refractive index of the adaptive lens.

## 5. Experiment

**Figure 12:**
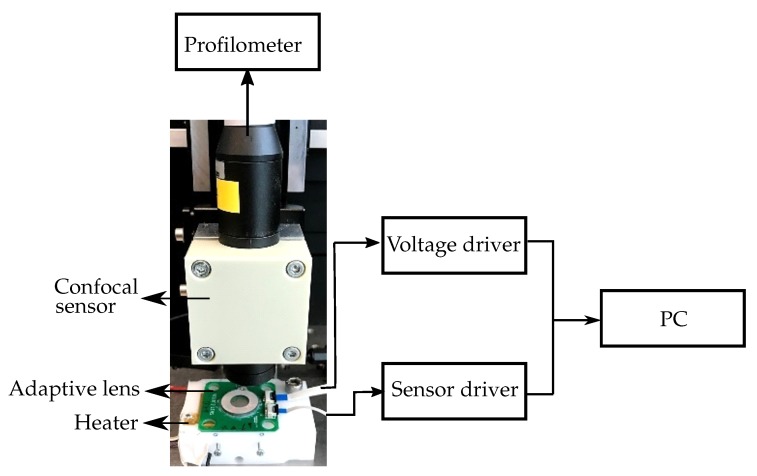
Experimental setup to characterize the adaptive lens at applied voltages and higher temperatures.

Furthermore, to verify the simulation results, we characterized the adaptive lens at different applied voltages and temperatures [7]. The adaptive lens integrated with a pressure sensor and a temperature sensor, to compensate for the non-linear piezoelectric hysteresis and the fluid thermal expansion effect [7,26], was used in the characterization. Figure 12 shows the block diagram of the experimental setup used to characterize the adaptive lens.

A sinusoidal voltage was applied to the piezoelectric actuator as was assumed in the simulation. A voltage driver was used to limit the negative voltage to −40 V, and the positive voltage to 140 V. A sensor driver was used to measure the output from the temperature and pressure sensor. The adaptive lens was mounted on a heater, which was used to heat the adaptive lens to the required temperatures. The membrane deformation was measured using a profilometer connected to a confocal displacement sensor providing a resolution of 110 nm. During the characterization, the adaptive lens was actuated and the corresponding applied voltage, membrane deformation, sensor outputs were measured simultaneously. The characterization was repeated with the adaptive lens set to higher temperatures. The refractive power was subsequently calculated from the measured membrane surface and then defined as a function of both the internal fluid pressure and the temperature. The measurements show the refractive power varying from $-16m^{-1}$ to $+17m^{-1}$ at 25 °C, and from $-15m^{-1}$ to $28m^{-1}$ at 75 °C.

**Figure 13:**
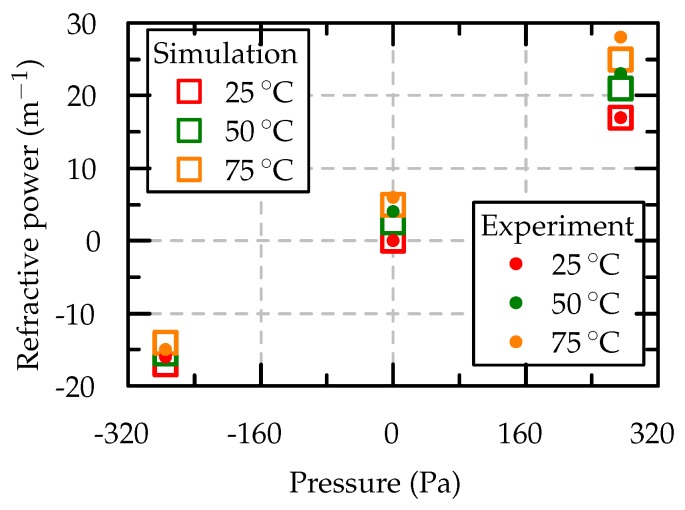
Comparison of the measured and simulated results of refractive power.

The simulated and measured refractive power of the adaptive lens at 25 °C, 50 °C and 75 °C with different applied voltages are compared in Figure 13. The temperature drift of the refractive power in the positive direction is higher than that in the negative direction because of the superposing effects of the thermal expansion of the fluid, which contribute to positive drift, and the increased actuator deflections at higher temperatures, which contribute to both positive and negative drift [7,19]. Hence, the superposed effect causes a net positive drift.

## 6. Conclusions

Our simulation model successfully couples the piezoelectric physics with the laminar flow and heat transfer physics modules. Both prove that the adaptive lens was simulated at different voltages and temperatures to determine the actuator deflection, the fluid pressure, and the refractive power. The simulated results are in close agreement with the experimental results. The adaptive lens can vary the refractive power from $-16m^{-1}$ to $17m^{-1}$ at 25 °C and from $-15m^{-1}$ to $28m^{-1}$ at 75 °C. With this validation, we can now use our model reliably for further geometric optimization of our adaptive lens. Furthermore, the simulation model could be extended to also model the piezoelectric hysteresis and change in piezoelectric coefficients with the temperature.

## Figures and Tables

**Table 1:** The materials of the adaptive lens components used in the simulation model.

Component	Material	Density (kg m${}^{-3}$)	Young’s Modulus (Pa)	Thermal Expansion Coefficient (K${}^{-1}$)
Piezoelectric actuator	Piezo PZT-5H [19]	7500	37 × 10^9^	1 × 10^−5^
Flexible membrane, rim	Polydimethylsiloxane [20]	1020	2 × 10^6^	3 × 10^−4^
Fluid	Fomblin Y [21,22]	1880	-	2.1 × 10^−4^
Substrate	Glass (quartz)	2210	50 × 10^9^	4 × 10^−5^

**Table 2:** The thickness of the adaptive lens components.

Component	Thickness (mm)
Membrane	0.2
Piezoelectric actuator	0.2
Rim	0.8
Substrate	1

## Abbreviations

The following abbreviations are used in this manuscript:

PDMS	Polydimethylsiloxane
FSI	Fluid-structure interaction
FEM	Finite-element method
CNC	Computer numerical control
PCB	Printed circuit board
